# Patched-Related Is Required for Proper Development of Embryonic *Drosophila* Nervous System

**DOI:** 10.3389/fnins.2022.920670

**Published:** 2022-08-23

**Authors:** Carmen Bolatto, Sofía Nieves, Agustina Reyes, Silvia Olivera-Bravo, Verónica Cambiazo

**Affiliations:** ^1^Developmental Biology Laboratory, Histology and Embryology Department, Faculty of Medicine, Universidad de la República (UdelaR), Montevideo, Uruguay; ^2^Cell and Molecular Neurobiology Laboratory, Computational and Integrative Neuroscience (NCIC) Department, Instituto de Investigaciones Biológicas Clemente Estable (IIBCE), Montevideo, Uruguay; ^3^Bioinformatic and Gene Expression Laboratory, Institute of Nutrition and Food Technology (INTA)-Universidad de Chile and Millennium Institute Center for Genome Regulation (CRG), Santiago, Chile

**Keywords:** Patched-related, *Drosophila*, embryogenesis, Hedgehog, neurodevelopment

## Abstract

*Patched-related* (*Ptr*), classified primarily as a neuroectodermal gene, encodes a protein with predicted topology and domain organization closely related to those of Patched (Ptc), the canonical receptor of the Hedgehog (Hh) pathway. To investigate the physiological function of Ptr in the developing nervous system, *Ptr* null mutant embryos were immunolabeled and imaged under confocal microscopy. These embryos displayed severe alterations in the morphology of the primary axonal tracts, reduced number, and altered distribution of the Repo-positive glia as well as peripheral nervous system defects. Most of these alterations were recapitulated by downregulating *Ptr* expression, specifically in embryonic nerve cells. Because similar nervous system phenotypes have been observed in *hh* and *ptc* mutant embryos, we evaluated the Ptr participation in the Hh pathway by performing cell-based reporter assays. Clone-8 cells were transfected with *Ptr*-specific dsRNA or a *Ptr* DNA construct and assayed for changes in Hh-mediated induction of a luciferase reporter. The results obtained suggest that Ptr could act as a negative regulator of Hh signaling. Furthermore, co-immunoprecipitation assays from cell culture extracts premixed with a conditioned medium revealed a direct interaction between Ptr and Hh. Moreover, *in vivo Ptr* overexpression in the domain of the imaginal wing disc where Engrailed and Ptc coexist produced wing phenotypes at the A/P border. Thus, these results strongly suggest that Ptr plays a crucial role in nervous system development and appears to be a negative regulator of the Hh pathway.

## Introduction

Patched-related (Ptr) is a transmembrane sterol-sensing domain (SSD) protein that is expressed in different insect and vertebrate species (Zúñiga et al., 2009). Ptr topology and domain organization are similar to those of the *Drosophila* segment polarity protein Patched (Ptc, Nüsslein-Volhard and Wieschaus, 1980). However, Ptr shows a distinctive expression pattern during embryogenesis (Bolatto et al., 2015). Ptr protein was first described in *C. elegans* (Kuwabara et al., 2000; Michaux et al., 2000), where functional studies suggested that the many Ptr proteins are involved in cell growth, patterning, and molting (Kuwabara et al., 2000). On the other hand, the single *Drosophila Ptr* gene (CG11212) was originally identified during a subtractive hybridization screening designed to identify genes differentially expressed at the beginning of gastrulation (Zúñiga et al., 2009). In addition, our biochemical analysis revealed that Ptr was associated with embryo membranes, and immunohistochemistry allowed us to localize it in the growing plasma membranes of *Drosophila* blastoderms (Pastenes et al., 2008). We also demonstrated that, during the late embryonic stages, Ptr accumulates in hemocytes (Bolatto et al., 2015), the phagocytic cells that are closely linked to the *Drosophila* developing nervous system (NS). Hemocytes are relevant cells because they participate in two main processes necessary for the NS condensation, the removal of cellular debris, and the deposition of extracellular matrix molecules (Hortsch et al., 1998; Sears et al., 2003; Olofsson and Page, 2005).

Regarding the study of the Ptr presence and function in the NS, a previous report (Furlong et al., 2001) classified *Ptr* as a gene mainly expressed in the neuroectoderm. For their part, Zhao et al. (2008) observed widespread macroscopic defects in the pattern of dendritic and axonal projections in neurosecretory neurons upon the insertion of a transposable element upstream of *Ptr*, likely because of disrupted neurite pathfinding. The same authors also demonstrated that a lack of function in Ptr results in the loss of bilateral asymmetry in the main portions of the normal neuritic arbor and the formation of ectopic or mistargeted neurites in the adult central nervous system (CNS) immediately prior to the emergence from the puparium (Zhao et al., 2008). Thus, Ptr loss disrupted the branching pattern in neurons that are essential for successful head eversion (Park et al., 2003). In spite of this crucial event, the only effects reported so far for Ptr at the NS level refer to the morphological changes probably induced by the disruption of the neuritic guidance.

The fact that Ptr is a transmembrane protein with similar topology to Ptc suggests that Ptr could act as a receptor in the axonal growth and/or pathfinding processes during NS development, regulating the availability of extracellular signals as Hedgehog (Hh). The possibility of an additional/alternative receptor in the Hh pathway has been previously suggested in other reports (Méthot and Basler, 2001; Torroja et al., 2005), including a proposed direct interaction between the secreted Hh and its receptor/s to promote cell-cell signaling (Bürglin, 1996; Aspöck et al., 1999) by depending either on Smoothened (Smo) as suggested for the majority of organisms or on a Smo-independent pathway as shown in *C. elegans* (Zugasti et al., 2005). In *Drosophila*, there are also examples of Hh acting in processes using non-canonical mechanisms, some of which are linked to developmental cell migration and guidance, such as germ and glial cell migrations and axon guidance (Araújo, 2015).

Interestingly, *hh* and *ptc* mutant embryos showed severely altered CNS phenotypes. In the former, many midline cells died, and the remaining surviving cells did not differentiate (Jacobs, 2000; Bossing and Brand, 2006; Watson et al., 2011). Meanwhile, in *ptc* mutant embryos, very few commissures could be formed, and this was mainly attributed to neuronal misspecification and loss (Patel et al., 1989; Merianda et al., 2005). In addition, the axonal defects observed in both null phenotypes could be attributed to alterations in the chemoattraction and/or chemorepulsion signals that are required for proper axon guidance (Ricolo et al., 2015). The same authors also showed that Hh is involved in axonal guidance during embryonic stages, acting in the ventral nerve cord (VNC) midline through a non-canonical Ptc-dependent pathway.

Considering the existing evidence, we decided to further study the Ptr function during the developing NS by analyzing whether embryos that lack or downregulate *Ptr* expression in embryonic neural cells show mutant NS-altered phenotypes that can be distinguished from wild-type embryos. Expected NS alterations include disruption of the NS structure, axon tract collapses or misrouting, fasciculation defects or alterations in the number or distribution of neurons.

In addition to characterize the Ptr role during embryogenesis, here, we also provide genetic, biochemical, and molecular evidence indicative of the Ptr involvement in the Hh pathway, probably acting either as an accessory component of the pathway or as an alternative receptor for the Hh signaling during NS development.

## Materials and Methods

### *Drosophila* Strains and Genetic Manipulations

All *Drosophil*a stocks were maintained and crossed at 25°C according to standard procedures. To obtain a more efficient gene silencing, the crosses involving RNAi experiments were carried out at 29°C (Brand et al., 1994). The stock en-GAL4, UAS-mCD8GFP/S-T (line *en*-GAL4), was kindly donated by A. Glavic (University of Chile, Chile), while the other GAL4-drivers were obtained from the Bloomington *Drosophila* Stock Center (BDSC, USA). The drivers used to express dsRNA*Ptr* were P{GAL4::VP16-nos.UTR}MVD2 (line *nanos*-GAL4) and w[^*^]; P{w[+mW.hs]=GawB}insc[Mz1407] (line 8751), whereas the line used to overexpress *Ptr-*mCherry was y[1] w[^*^]; P{w[+mW.hs]=en2.4-GAL4}e16E (line 30564). To perform the control crosses w[^*^]; P{w[+mC]=UAS-lacZ.B}melt[Bg4-2-4b] (line UAS-*lacZ*) was used. The original *Ptr* mutant line (*Ptr*^23*c*^line) balanced over *CyO, kr*-GFP balancer (Bolatto et al., 2015) was re-balanced using *CyO, twist*-GAL4, UAS-GFP donated by R. Cantera (Instituto Clemente Estable, Uruguay) to obtain the line *Ptr*/*twi*-GFP.

### Determination of Hatching Rate

Adult flies of the *Ptr*/*twi*-GFP null mutant line were placed in cages for embryo collection for 4 h at 25°C. Eggs laid on grape juice agar plates were left for an additional 18-24 h. After that, the embryos were dechorionated with 50% commercial bleach solution in PBST (0.05% Triton X-100 in PBS), classified as GFP positive/negative and hatched/not hatched, and counted under a Nikon SMZ-10A stereomicroscope (Tokyo, Japan) using NIGHTSEA^®^ fluorescence viewing systems (PA, USA) with a royal blue filter. Light and fluorescence images were obtained using a Discovery V8 stereomicroscope (Zeiss, Oberkochen, Germany).

### Embryo Immunostaining

*Patched-related* null mutant embryos of stage 15/16 were collected from grape juice agar plates, dechorionated (50% commercial bleach solution in PBST) and selected by negative GFP expression. Selected embryos were fixed in a 1:1 (v/v) mixture of n-heptane and 4% paraformaldehyde (PFA) for 30 min, and their devitellinization was performed with a slow fixation procedure (Sullivan et al., 2000) using 4% PFA (Sigma-Aldrich, MO, USA) instead of 3.7% formaldehyde. The embryos were stored in methanol at -20°C until used. To perform the immunostaining, the embryos were rehydrated in PBSTA (1% BSA in 0.05% Triton X-100 in PBS) and incubated 30 min in a blocking buffer (1% BSA in 0.1% Triton X-100 in PBS). The same blocking buffer was used during the antibody incubation. Antibodies employed were: mouse anti-22C10 [1:50 22C10, Developmental Studies Hybridoma Bank (DSHB, IA, USA)] or mouse anti-FasII (1D4 anti-Fasciclin II, DSHB, diluted 1:30) or mouse anti-Repo (1:20 8D12 anti-Repo, DSHB). These antibodies were incubated together with lectin from *Arachis hypogaea* (peanut or PNA) biotin conjugate (1:200, Sigma-Aldrich) or rat anti-elav (1:50 Rat-Elav-7E8A10 anti-elav, DSHB). After overnight incubation at 4°C, embryos were washed three times with PBST and one time with a blocking buffer (15 min each) and then incubated 2 h with anti-mouse Alexa Fluor 488 (1:800, Molecular Probes, OR, USA) and anti-rat Alexa Fluor 546 (1:800, Molecular Probes) or Streptavidin Alexa Fluor 555 (1:800, Molecular Probes). After 4 washes (15 min each) with PBST, the embryos were mounted using glycerol 80% in Tris-HCl (1.5 M, pH 8.8) in a chamber made with two coverslips. The use of this chamber allowed us to rotate the embryos to locate the ventral side upward. Digital images were taken using an Olympus FV300 laser scanning confocal microscope at 1,024 × 1,024 or 2,048 × 2,048 resolution, ensuring that objective lens, brightness, laser intensities, and gain were standardized to ensure the maintenance of acquisition parameters in the samples of each experimental condition.

### Cell Culture and Generation of Conditioned Medium

Cultures of clone-8 (cl-8) cells (derived from the *Drosophila* wing imaginal disc) were performed as described at the *Drosophila* RNAi Screening Center website (http://www.flyrnai.org/DRSC-PRC.html). The HhN conditioned medium was made by incubating S2-HhN-transfected cells (generously donated by P. Beachy, Stanford University), with a cl-8 medium plus 0.5 mM CuSO_4_ for 48 h. The control medium was made by incubating S2 cells in the same culture medium and under the same conditions.

### Immunostaining of cl-8 Cells

Cells were fixed with 4% PFA (20 min, RT), permeabilized with PBS containing 0.1% saponin for 15 min, and then blocked with PBS plus 5% BSA and 0.1% saponin for 45 min prior to incubation with the primary antibody mouse monoclonal anti-V5 (1:500, Sigma-Aldrich, MO, USA). The cells were washed three times in PBS with 0.1% saponin and incubated with the secondary antibody anti-mouse Alexa Fluor 546 (1:800, Molecular Probes). Zeiss Confocal images were collected using the Confocal Laser Scanning Microscope-510 META.

### dsRNA Synthesis

Pairs of primers encoding a T7 promoter sequence and gene-specific sequences were used to amplify a product from a single exon using a genomic DNA template or cDNA. These PCR products were the templates for *in vitro* dsRNA synthesis using T7 RNA polymerase (Ambion, TX, USA). See Supplementary Material for the table of primers used.

### Constructs for Cell-Based Reporter Assays

The *ptc*-luciferase, *Renilla*, and *dally-like* constructs were kindly donated by Dr. P. Beachy. The *ptc*-luciferase (the *ptc* promoter −758 to +130 fragment) was generated by PCR and subsequently cloned into the MluI and HindIII sites of the pGL2-Basic firefly luciferase reporter vector (Promega, Madison, USA); the *Renilla* construct was pRL-CMV (for constitutive *Renilla* luciferase expression under CMV promoter control), and *dally-like*-3xFlag was inserted into pActSv (the cloning sites can be found by sequencing with Fw GAC ACA AAG CCG TTC CAT and reverse TTT GTC CAA TTA TGT CAC) (Chen et al., 1999).

*Ptr*-V5 and the control construct of overexpression experiments in cell reporter assays were obtained in Dr. V. Cambiazo's laboratory. The CG11212 fragment (residues 1–1,129) was generated by PCR and cloned into pMT/V5-His-Topo (Invitrogen, CA, USA) (Zúñiga et al., 2009). The control vector for overexpression experiments was pUASpEGFPc1 (Megraw et al., 2002). This vector derives from the pUASP vector, encoding for an enhanced green fluorescent protein and expressed in *Drosophila* cells.

### Luciferase Reporter Assays

dsRNA (2 μg) and/or *Ptr*-V5 or a control vector construct (2 μg) and/or vectors that express *ptc*-luciferase, *dally-like*, and copia-*Renilla* control (to normalize transfection efficiency) were transfected into cultured cl-8 cells using the Calcium Phosphate Kit (Invitrogen) according to Chen et al. (1999) and manufacturer instructions. Transfection was followed by an incubation of 72 h to allow for protein turnover and degradation of targeted mRNA. Transfected cells cultured in 24-well plates were then split by repeated pipetting and seeded into the control medium or into the HhN-conditioned media, and the cells were incubated for additional 24 h. When required, 0.5 mM CuSO_4_ was added to the cell medium to induce Ptr protein expression. Then, the cells were lysed, and the luciferase activity in the lysates was measured using the Dual-Luciferase Reporter Assay Kit (Promega), which enabled the sequential measurement of firefly and *Renilla* luciferase. Reporter firefly luciferase activity (L) was normalized to the *Renilla* luciferase activity (R) and expressed as the L/R ratio. Fold induction upon stimulation with Hh following transfection of pooled dsRNAs was determined by dividing the L/R ratio obtained in the presence of Hh by the L/R ratio measured in the absence of Hh (basal reporter activity). In all experiments, dsRNA targeting the *B. subtilis lys* gene for diaminopimelate decarboxylase and/or a vector expressing GFP was used as a control.

### Immunoprecipitation Assays

Cell extract was obtained by lysing the content of three 60 mm culture dishes of cl-8 cells expressing Ptr-V5 with a 600 μl cold lysis buffer [20 mM Tris, pH 7.5, 150 mM NaCl, 1 mM EDTA, 1 mM EGTA, 1% Triton X-100, and Sigma Fast, Protease Inhibitor Cocktail (Sigma-Aldrich)]. The lysate was centrifuged for 10 min at 16,000 g at 4°C and the supernatant stored at -80°C. The S2-HhN-conditioned medium was lyophilized and concentrated 10-fold. Protein determination was carried out using the Bradford method (Fermentas, MA, USA). The input sample for the immunoprecipitation was obtained by incubating equal parts of the Ptr-V5 cl-8 cell extract and the S2-HhN-conditioned medium (each one contained 1 mg/ml of protein) over 16 h at 4°C. The premix (input sample) was immunoprecipitated with 1 μg of a mouse anti-V5 antibody (Santa Cruz Biotechnology, TX, USA) adsorbed to 25 μl of goat anti-mouse IgG Dynabeads (Invitrogen). For the control, Dynabeads were incubated with an alternative mouse antibody (anti-c-Myc, Santa Cruz Biotechnology). Beads were washed three times and proteins eluted in a Laemmli sample buffer. The input sample and the eluents of anti-V5 and anti-c-Myc immunocomplexes were analyzed by Western blotting as described below.

### Western Blotting

To detect the presence of HhN or Ptr-V5, the immunocomplex was separated by SDS-PAGE and transferred to a PVDF membrane for 2 h at 100 V. Before loading the gel, the samples were incubated with a Laemmli sample buffer for 30 min at 37°C (not boiled) to avoid the formation of aggregates that impede the uniform binding of SDS to the sample proteins. The membrane was blocked (1 h, RT), with 5% low-fat milk in Tris-buffered saline (pH 7.4) and cut at the level of pre-stained molecular weight, 43 kDa (Fermentas). The piece with lower molecular weight proteins was incubated overnight at 4°C with 1:100 rabbit anti-Hh (Santa Cruz Biotechnology), diluted in 1% low-fat milk, 0.05% Tween 20 in Tris-buffered saline (pH 7.4). The rest of the membrane was incubated with a mouse anti-V5 antibody (1:2,000) in similar conditions. To detect the primary antibodies and/or the immunocomplex, the membranes were incubated with anti-mouse or anti-rabbit secondary antibodies coupled to peroxidase (1:500, Thermo Fisher Scientific, MA, USA). The product reaction was revealed using the Supersignal West Pico chemiluminescent reagent (Amersham Biosciences, Bucks, United Kingdom) and the sensitive X-ray film (Amersham Biosciences).

### RNA Extraction and cDNA Synthesis

Total RNA was extracted from staged embryos (*N* = 10-15) or transfected cells using the RNA_WIZ_ reagent (Ambion). The samples were carefully homogenized in a 1.5 ml Eppendorf tube with 1 ml of RNA reagent using one insulin syringe. After 5 min at RT, 0.2 volumes of chloroform were added, and the samples were subjected to a cycle of shaking, incubation (10 min, RT) and centrifugation (13,000 g for 15 min, 4°C). The RNA rescued from the aqueous phase was precipitated with isopropanol/glycogen by centrifugation at 14,000 g at 4°C for 15 min. The precipitate was washed with 75% ethanol and centrifuged at 14,000 g at 4°C for 5 min. Finally, the total RNA was re-suspended in 30 μl of nuclease-free water. An average yield of 1 μg/μl of RNA was obtained. RNA integrity was verified by agarose gel electrophoresis (1.2% formaldehyde-agarose gel). The samples were treated with TURBO DNA-free DNase (Ambion) to remove contaminating DNA from the RNA. For qPCR, 1 μg of total RNA was used as a template for reverse transcription reactions to synthesize single strand (ss) cDNA using MMLV-RT reverse transcriptase (Promega) and oligo-dT primer (Invitrogen) according to standard procedures. A poly (A)-RNA was *in vitro* transcribed from the vector pGIBS-dap (ATCC 87486) and added to the embryo RNA samples prior to cDNA synthesis in a 1:1,000 ratio to be used as spike mRNA (Brand et al., 1994).

### cDNA Synthesis and Quantitative Real-Time PCR

qPCR amplifications and fluorescence detection were performed using the LightCycler^®^ 1.5 Instrument (Roche, Basel, Switzerland) and LightCycler^®^ FastStart DNA Master SYBR^®^ Green I (Roche). Reactions contained 100 ng of dscDNA or 50 ng of sscDNA. Primers were designed using Primer Premier 5.0 software (Palo Alto, CA, USA) and synthesized by Alpha DNA (Quebec, Canada). Primer sequences, annealing temperatures, and amplicon lengths are given in Supplementary Material. For each gene, a calibration curve was generated based on serial dilutions (101-102 pg/μL) of plasmid templates. The thermal cycle conditions were: denaturation at 95°C for 10 min, followed by 35 three-step cycles of template denaturation at 95°C with a 2 s hold, primer annealing at 68°C for 15 s, and extension at 72°C for 60 s/1,000 bp. The purity of amplified products was verified by melting curve analyses. Control reactions included a subset of PCR components lacking the cDNA template. The initial amount of transcript in each sample was calculated from the standard curve using the default (fit point/arithmetic) method of LightCycler Software Version 3.5 and normalized to the values of *actin*. Data represent the mean ± SEM of three replicates from three independent experiments.

### RNAi Vector Construction, Microinjection, and Generation of UAS-DsARN *Ptr* Lines

A region of the first exon of *Ptr* was generated by PCR using genomic DNA as a template with the primers indicated in Supplementary Table 1. To create the knockdown plasmid UAS-*Ptr*IR, the PCR product was inserted into the pWiz vector (*Drosophila* Genomics Resource Center, IN, USA) at each of the AvrII and NheI restriction sites, in opposite orientations (Lee and Carthew, 2003). Clones were confirmed by sequencing. *w*^1118^ embryos were injected with the UAS-*Ptr*IR construct at Genetic Services Inc. (MA, USA) according to standard protocols (Rubin and Spradling, 1982). Homozygous lines were generated with standard balancer chromosomes.

### Generation of the UAS-*Ptr*-mCherry Lines

The cDNA for the *Ptr* gene was subcloned into a pUAST vector by a standard protocol and sequenced. Transgenic plasmids (with the UAS sequence and the mCherry tag fused to the C-terminal end of *Ptr*) were mixed with 100 μg/ml of helper plasmid (P {Δ2-3}) and injected into eggs [w1118] as described by Rubin and Spradling (1982). Surviving F0 males or females were individually crossed with virgin females [w1118] or males [w1118]. The progeny of these crosses (F1 generation) with colored eyes was used to establish transgenic lines through crossings with lines containing different balancer chromosomes. VectorBuilder Inc. (IL, USA) cloned the designed plasmid (pUASTattB-5 × UAS/mini_Hsp 70 > {CG11212}/mCherry) and tested its quality. BestGene Inc. (CA, USA) microinjected the embryos and balanced the transgenic lines.

### Analysis of Adult Wings

Wings from male and female adult flies were removed from the thorax and incubated in a washing buffer (PBS and 0.1% Triton X-100) separately by gender. A paintbrush was used to mount the wings on a slide with 80% glycerol in PBS (Gault et al., 2012). Light images of the wings were obtained with a Nikon Eclipse E400 microscope at 10X magnification. Quantitative image processing was carried out using Fiji ImageJ software (NIH). To do this, we delimited the areas to be measured (the total wing area and the L3-L4 intervein area), with the aid of the Polygon selection tool, and the selected pixels were counted with the Analyzed option. In order to make an appropriate comparison, the quotient L3-L4 intervein area/total wing area of each wing was determined and graphed. Mean ± SEM were represented. Around 30 males and 30 females for each cross were measured.

### Statistical Studies

Data are expressed as the mean ± SEM. Statistical tests used were ordinary one-way ANOVA followed by the Tukey's *post-hoc* comparisons or the unpaired two-tailed Student's *t-*test (GraphPad Prism 5.0, Mac OS X version). Statistical significance was determined at *p* < 0.05. The number of asterisks-^*^, ^**^, ^***^–indicates *p* < 0.05, *p* < 0.01 or *p* < 0.001, respectively. Normal distribution of the data was tested using GraphPad Prism 5.0, Mac OS X version.

## Results

### Absence of *Ptr* Elicits Alterations in Some Components of the Developing Central and Peripheral NS

Because previous studies have classified the *Ptr* coding gene sequence as a neuroectodermal gene (Furlong et al., 2001), we decided to analyze whether *Ptr* null mutants exhibit an embryonic CNS with disturbed architecture. We analyzed late homozygous embryos (stages 15/16) that were characterized by the lack of GFP fluorescence. Only ≅19% of these embryos hatched (Supplementary Figure 1), strongly reinforcing the previous evidence (Bolatto et al., 2015) that Ptr is critical to *Drosophila* development. In up to 100% of the cases, the larvae did not reach adulthood under standard feeding conditions.

**Figure 1:**
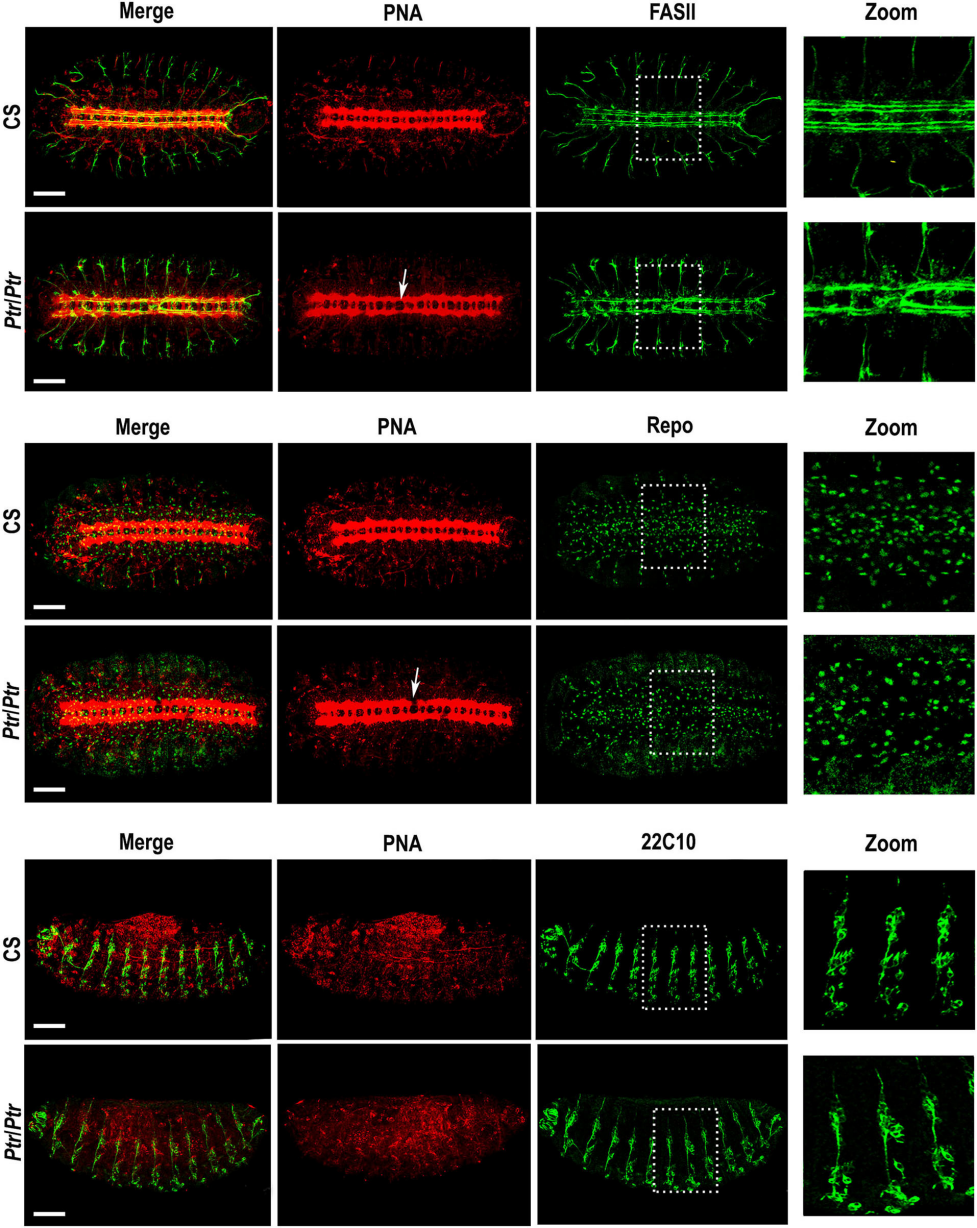
Phenotypic analysis of the *Ptr* null mutant in whole mount embryos. Z-stack immunofluorescences against PNA and FASII (the two upper rows), PNA and Repo (the two middle rows) or PNA and 22C10 (the two bottom rows). The analysis was carried out on *D. melanogaster* late embryos (stage 15/16). Arrows indicate the alterations in the primary tracts of *Ptr* null mutants that were evidenced with PNA. The right column shows a higher magnification of the alterations found inside the dashed boxes in each image. In all the figures, embryos are oriented anterior to the left. To observe the nervous system, the first four rows of images are ventral views of the embryo, whereas, in the last two rows, the ventral side of the embryo is down. Bars: 50 μm.

To perform this study, we employed PNA lectin to visualize the morphology of axon tracts in the VNC as shown by D'Amico and Jacobs (1995). In *Ptr* null mutant late embryos, we detected interruptions or alterations in the regular spaces delimited by VNC commissures and connectives tracts that were analyzed in detail by combining the biotinylated lectin with specific primary antibodies. These alterations were consistently found between abdominal segments A2-A6. When anti-FASII and PNA were combined, it became evident that the alterations previously observed with the lectin (Figure 1, white arrows in images of PNA staining) correlated with FasII-positive longitudinal axons that were over-migrating and re-crossing the midline (Figure 1, the two upper rows).

Anti-Repo and PNA co-labeling indicated the co-existence of the primary axonal tract interruptions and alterations in the number and/or distribution of Repo-positive glial cells (Figure 1, the two middle rows). In addition, the combined staining using anti-22C10 and PNA enabled us to establish that the lack of Ptr also produces failures at the PNS branching and distribution of neurons (Figure 1, the two bottom rows).

### *Ptr* Downregulation Using a Pan-Neural Driver Affects the Embryonic NS Structure

In a previous work, we reported differences in the number and distribution of migrating hemocytes in the *Ptr* null mutant (Bolatto et al., 2015). Considering that hemocytes are essential to NS embryogenesis (Sears et al., 2003), here, we aimed to know whether the morphological changes described above resulted directly from the lack of Ptr in the NS or indirectly by the reduced action of hemocytes. To elucidate this point, we expressed dsRNA of *Ptr*, specifically in the NS using a pan-neuronal driver (line 8751 of BDSC).

First, we generated a transgenic line that expressed an inverted repeat of the first exon of the *Ptr* sequence under the control of the UAS promoter in the vector pWIZ (UAS-dsRNA*Ptr*). To determine if the construct designed was effective in silencing the *Ptr* gene, the transgenic lines were crossed with the *nanos*-GAL4 driver to activate transcription of the hairpin-encoding transgene in the progeny. As a control, GAL4 drivers were crossed with UAS-*lacZ* flies. Using qPCR analysis, we determined that the amount of *Ptr* mRNA in *nanos*>UAS-dsRNA*Ptr* embryos was reduced by about 90% of the amount found in the control embryos (*nanos*>UAS-dsRNA*Ptr*) (Supplementary Figure 2).

**Figure 2:**
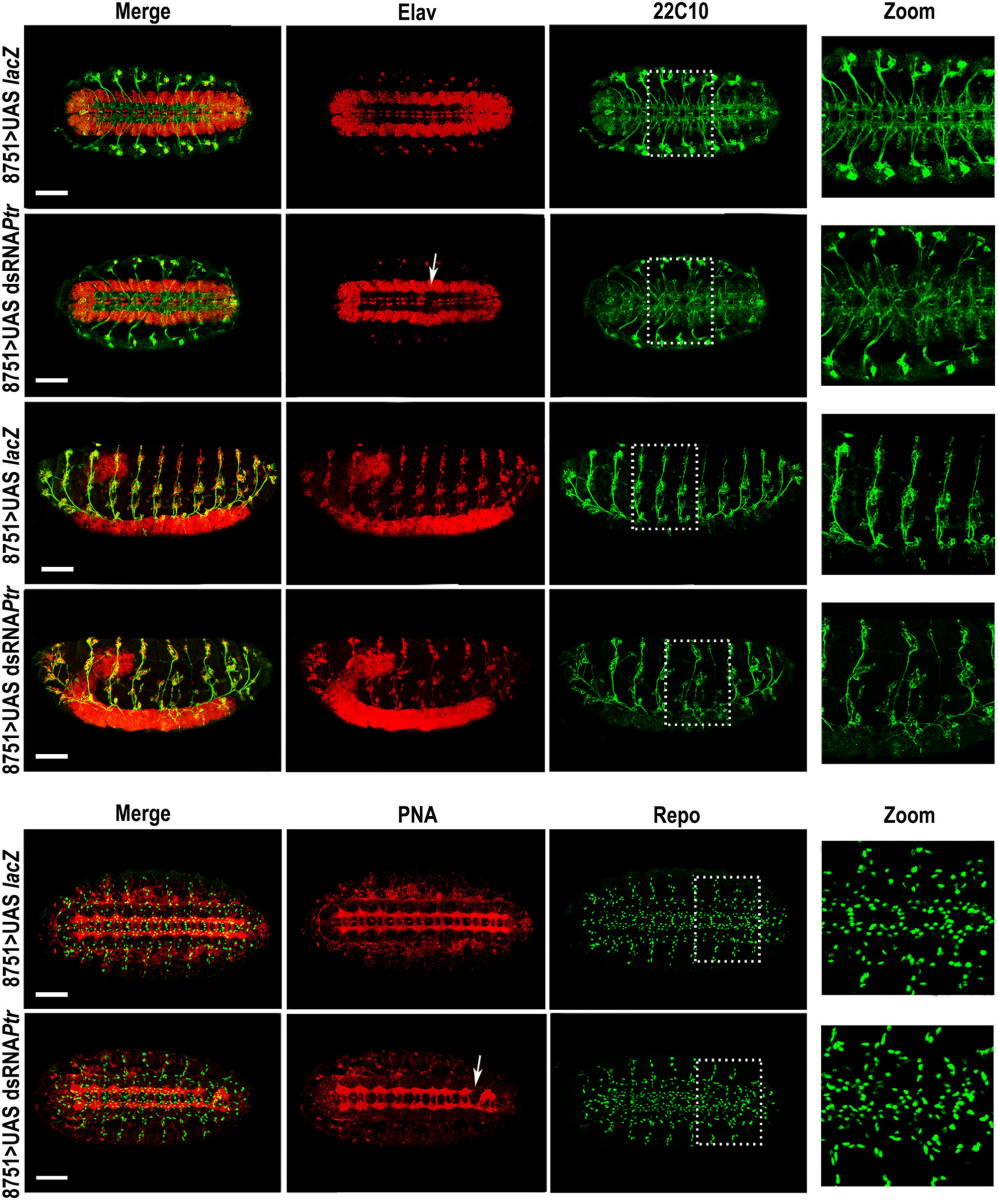
Downregulation of *Ptr* specifically driven to nervous system recapitulates several morphological changes found in *Ptr* null mutants. Z-stack immunofluorescence against Elav and 22C10 (the four upper rows) or PNA and Repo (the two bottom rows). The analysis was carried out on *D. melanogaster* late embryos (stage 15/16). Arrows indicate the alterations seen in the primary tracts. The right column shows a higher magnification of the alterations found inside the dashed boxes in each image. In all the figures, embryos are oriented anterior to the left. To observe the nervous system, most images are ventral views of the embryo, except the third and fourth rows that show lateral views where the ventral side of the embryo is down. Bars: 50 μm.

Regarding immunostaining, we decided to use rat anti-elav (red in Figure 2), a pan-neuronal marker for most cells in the CNS and PNS, to evidence the VNC distortions (white arrows). The use of this antibody together with anti-22C10 demonstrated that axon crossovers occurred, recapitulating the phenotype observed in the *Ptr* null mutant. In addition, the lateral view of the embryo revealed that the crosslinking resulted in the presence of fewer axons at the PNS, as well as important disorganizations in the peripheral neurons (mis-migrating and morphological irregularities in lch5 chordotonal neurons) (Figure 2). Table 1 summarizes the penetrance of *Ptr* null mutants and NS knockdown phenotypes.

**Table 1:** Penetrance of SN phenotypes.

**Phenotype**	***CS***	***Ptr/Ptr***	***8751>UAS-lacZ***	***8751>UAS-dsRNAPtr***
Longitudinal axon defects	0% (40)	20% (30)	0% (28)	30% (30)
Repo-Positive glial defects	0% (30)	^≅^43% (28)	0% (25)	^≅^53% (30)
PNS organization defects	0% (30)	^≅^37% (30)	0% (25)	^≅^44% (32)

*Penetrance is defined as the percentage of embryos that present the phenotype. The total number of embryos analyzed for each condition is shown in parentheses. PNS, peripheral nervous system*.

**Figure 3:**
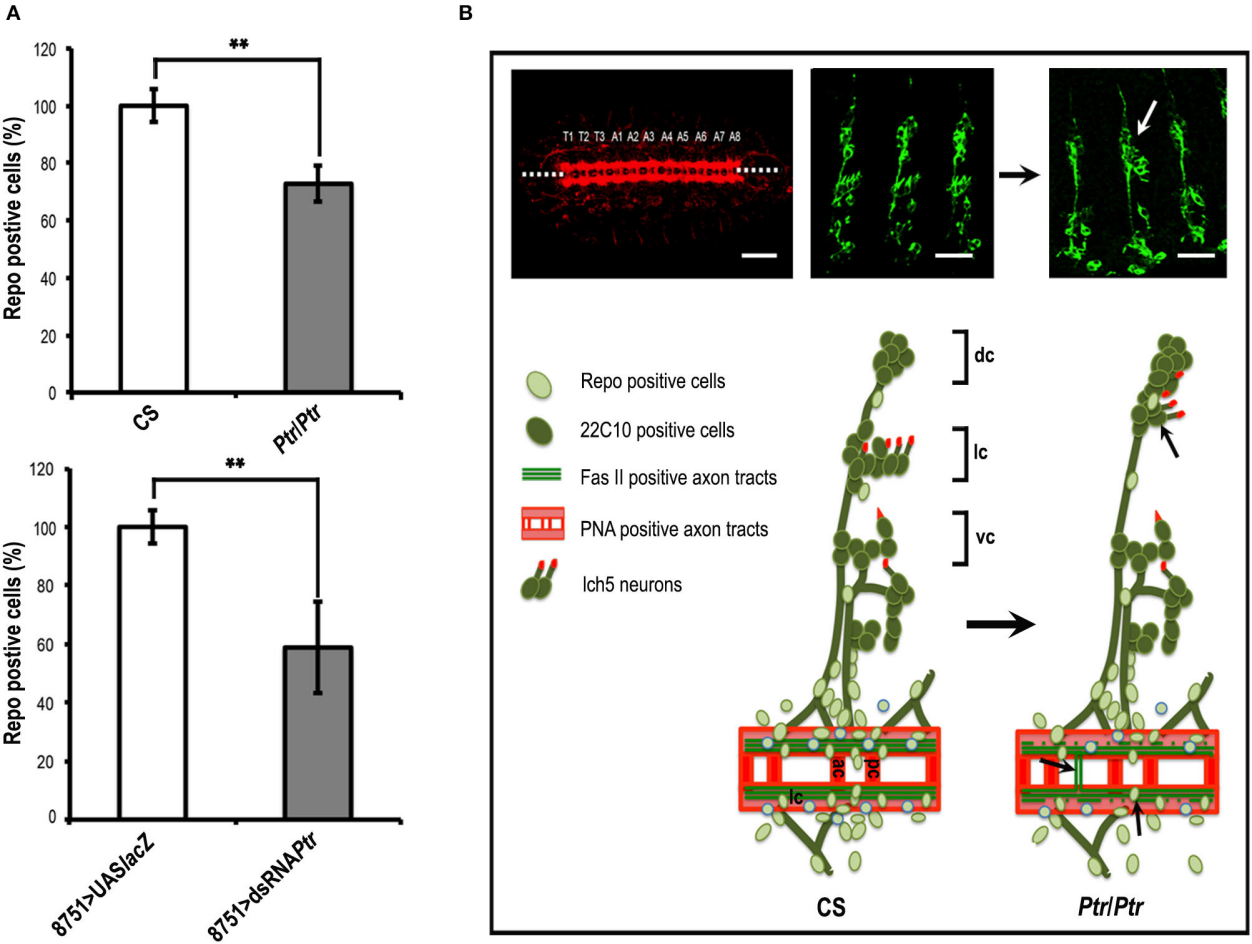
The absence of Ptr produces morphological irregularities at the nervous system level. **(A)** Quantitative analysis of Repo-positive glial cells in CS and *Ptr* mutant (the upper graph) and UAS-*LacZ* and dsRNA *Ptr* (the bottom graph) embryos at the 15/16 stage. Data represented is the mean ± SEM. (**) indicates *p* < 0.01. The results showed ≅30 and 40% fewer Repo-positive cells in the affected abdominal segments (A2–A6) of mutant and downregulated embryos. **(B)** Ventral and lateral images of late *Drosophila* embryos. The left image shows a ventral view of a late embryo stained with PNA lectin that labels CNS axonal tracts. The segmental CNV-repeated pattern is specified as T1–T3 for thoracic segments and A1–A8 for abdominal segments, as in Ivanov et al. (2004). Dotted lines indicate the embryo midline. Middle and right images show lateral views of CS and *Ptr* null mutant late embryos stained with 22C10 to visualize neuronal morphology and axonal projections. The white arrow indicates an evident lch5 neuronal miss-migration. The lower illustrations schematize the main PNS characteristics detected when comparing CS and *Ptr* null mutant embryos. Black arrows indicate the miss-migration of neurons, axon midline crossovers, and reduction of Repo-positive glia. Abbreviations in CNS: ac, anterior commissure; pc, posterior commissure; lc, longitudinal connectives. Abbreviations in PNS: dorsal (dc), lateral (lc) and ventral (vc) clusters, respectively. Bars: 50 and 75 μm for PNA and 22C10 staining, respectively.

Concerning Repo-positive cells, our results showed significant reductions in both *Ptr* null mutant (≅27%) and *Ptr* downregulation (≅41%), specifically in the NS (Figure 3A). A summary of the differences found in the *Ptr* null mutant compared to the NS wild-type embryos is shown in Figure 3B. Given that *Ptr* downregulation produces similar alterations, it indicated that Ptr has a role at the NS level that is relevant for its proper organization.

### Ptr Functions as a Negative Component of the Hh Pathway in cl-8 Cells

The important topological similarities between Ptr and the Hh pathway components, Ptc and Dispatched (Burke et al., 1999), prompted us to evaluate whether Ptr could be involved in the Hh signaling pathway. Moreover, previous reports have indicated that some of the genes associated with the Hh signal transduction pathway also develop mutant NS phenotypes, suggesting a link between the pathway and the NS developmental process (Patel et al., 1989; Merianda et al., 2005; Koizumi et al., 2007). Thus, we used a cultured cell assay developed by Philip Beachy's group (Chen et al., 1999; Lum et al., 2003), which has successfully identified new components of the Hh pathway (Yao et al., 2006). This system is quantitative and specific for cellular response because the addition of exogenous Hh, through a conditioned medium, makes the assay independent of ligand synthesis or distribution (Lum et al., 2003).

**Figure 4:**
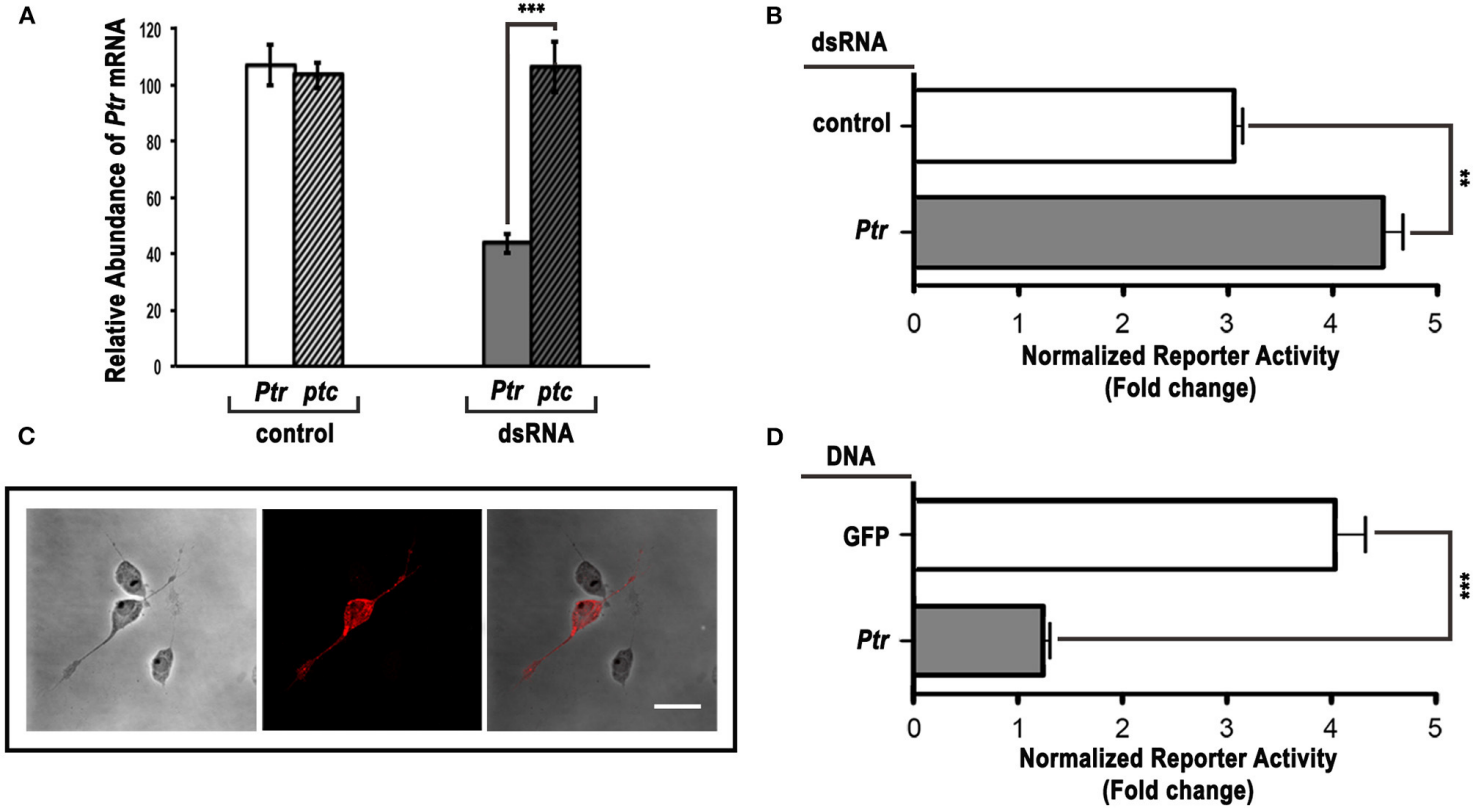
Ptr can act as a negative regulator of the Hh pathway in cl-8 cells. **(A)** The *Ptr*-specific dsRNA efficiently reduced the level of *Ptr* transcripts. Downregulation was specific for the target *Ptr* gene, and no changes were detected in the *ptc* expression level. **(B)** dsRNA targeting *Ptr* moderately enhanced the responsiveness to Hh when compared to the control dsRNA. **(C)** The Ptr-V5 overexpression in cl-8 cells was verified by immunostaining using a specific anti-V5 antibody (red). **(D)** *Ptr* expression strongly reduced the response to the Hh signal. As a control, a construct expressing GFP was transfected into cl-8 cells. In **(B,D)**, white bars are controls and gray bars are the experimental condition, respectively. Data are presented as mean ± SEM (*n* = 3 per group). Analysis was performed using the unpaired two-tailed Student's *t*-test, **p < 0.01 and ***p < 0.001. Bar: 5 μm.

Since RNAi in *Drosophila* cultured cells is frequently used as a functional test of gene products with a known or predicted sequence (Chen et al., 1999; Lum et al., 2003; Yao et al., 2006), we treated cl-8 cells with control or *Ptr*-specific dsRNA and assayed for changes in Hh-mediated induction of a Hh pathway-responsive luciferase reporter (a *ptc*-luciferase reporter construct) (Chen et al., 1999; Lum et al., 2003). Our results indicated that the transfection of *Ptr* dsRNA caused an exclusive reduction in *Ptr* mRNA levels (Figure 4A) and a significant increase in the response to Hh signaling (Figure 4B). On the other hand, transfection of cl-8 cells with a *Ptr* DNA construct to obtain higher levels of Ptr protein (Figure 4C) produced a strong and opposite effect on the Hh pathway activity (Figure 4D). The observed effect suggested that normal levels of Ptr in cl-8 cells could act as a limiting factor in the response to Hh signaling. These results also suggested that Ptr can modulate the response to Hh, and the outcome depends on cellular Ptr levels.

**Figure 5:**
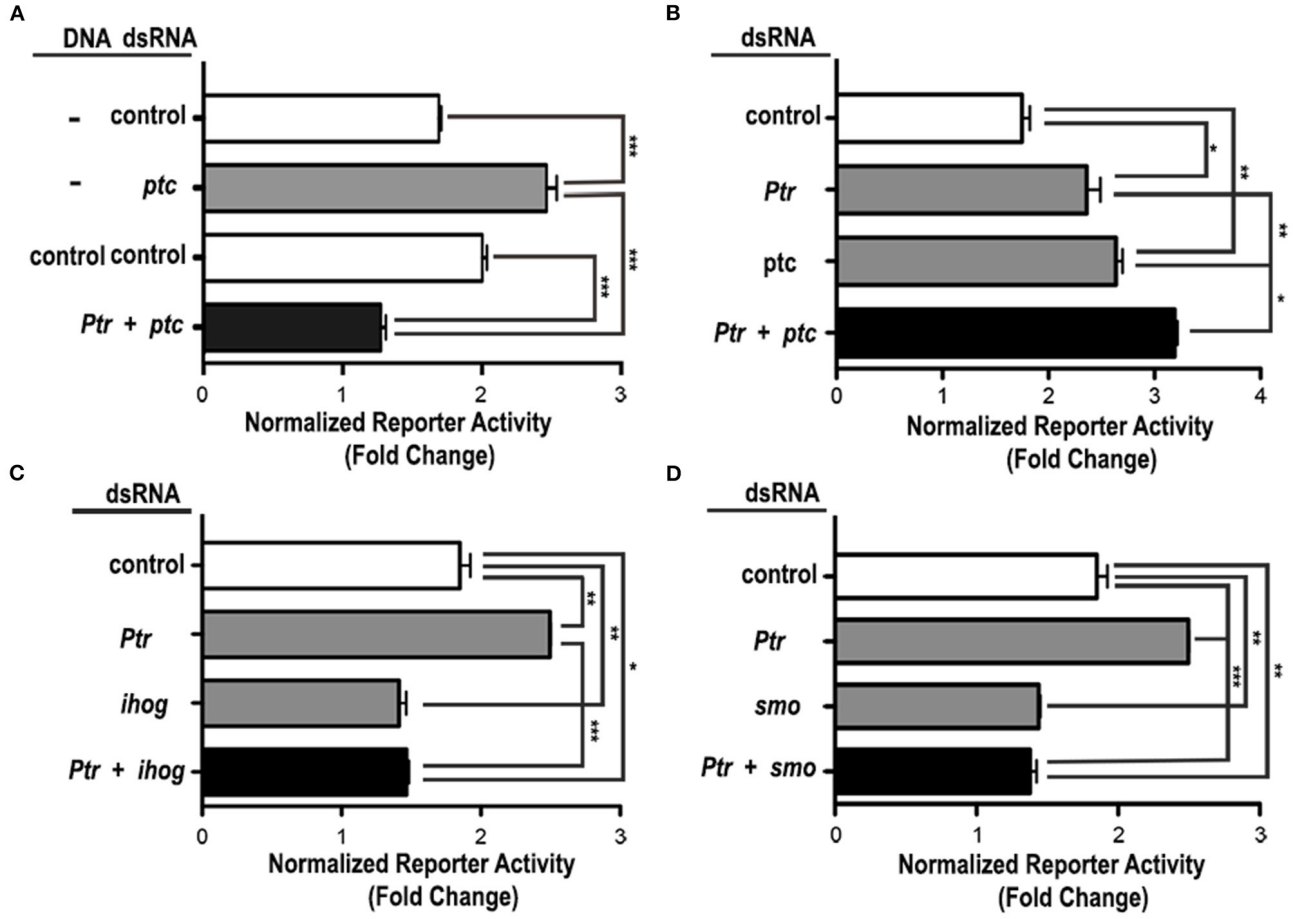
Ptr functions downstream or at the same level as Ptc in the Hh signaling cascade. **(A)** The enhanced response to the Hh signal caused by the knockdown of *ptc* can be suppressed by the co-transfection of a vector that expresses *Ptr*. dsRNAs targeting the *B. subtilis lys* gene and a construct for expression of GFP serve as controls. **(B)** The co-transfection of *Ptr* and *ptc* dsRNA produced an activation of the signaling pathway that was stronger than the activation obtained by the independent *knockdown* of each gene. *Ptr* functions seem to act independently on **(C)** iHog or **(D)** Smo. The transfection of *Ptr* dsRNA does not affect the activation level of the Hh pathway resulting from the knockdown of *ihog* or *smo* genes. In all the graphics, white bars are negative controls, gray bars are internal controls for the dsRNA and vector transfected, and black bars are the experimental condition for each set of experiments. Data are presented as mean ± SEM, *n* = 3 per group. Analysis was performed using one-way ANOVA with the Tukey *post-hoc* tests, *p < 0.05, **p < 0.01 and ***p < 0.001.

To investigate the role of the Ptr protein in Hh signaling related to Ptc, we performed luciferase reporter assays in cl-8 cells to downregulate *ptc via* dsRNA together with downregulation or overexpression of *Ptr*. The overexpression of *Ptr* not only suppressed the increase in the Hh pathway activity produced by *ptc* dsRNA but also reversed it, resulting in levels of the pathway activity lower than control (Figure 5A). Co-transfection with *ptc* and *Ptr* dsRNA produced activation of the signaling pathway stronger than the activation produced by independent transfection with dsRNAs targeting each gene (Figure 5B). Thus, these results strongly suggested that Ptr may act independently of Ptc.

To further investigate the mechanism of Ptr action on the Hh signal response, we examined the effect produced by cell co**-**transfection with the dsRNA of *Ptr* in combination with dsRNAs targeting other known pathway components. Simultaneous transfection of *Ptr* dsRNA and either *ihog* or *smo* dsRNA had a pathway activity similar to that obtained when *ihog* and *smo* are individually silenced (Figures 5C,D). These results suggested that Ptr acts on Hh signaling upstream of these two components (iHog and Smo) of the Hh signaling pathway. Taken together, these results indicated that Ptr could play a role in the regulatory mechanism of the Hh signal transduction. Complementary assays are required to evaluate whether Ptr would be fulfilling a role similar to that of Ptc, especially when considering the literature reports that iHog acts upstream of Ptc (McLellan et al., 2006; Yao et al., 2006; Camp et al., 2014).

### *In vitro* Binding of Ptr and Hh

**Figure 6:**
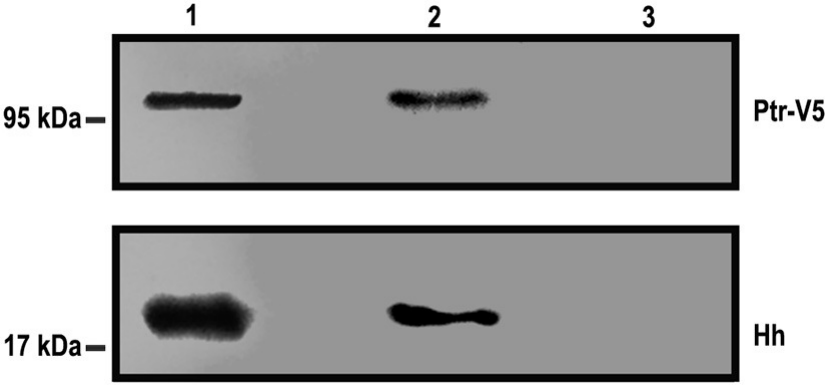
Hh can precipitate Ptr-V5. Hh and Ptr-V5 expression vectors were transfected into S2 and cl-8 cells, respectively. A premix of an S2-HhN-conditioned medium and a lysate of cl-8 cells overexpressing the *Ptr*-V5 fusion protein (an input sample) was immunoprecipitated with anti-V5 or anti-c-Myc antibodies, each one adsorbed into goat anti-mouse IgG Dynabeads. The input sample (Lane 1) and the eluents from immunoprecipitation with anti-V5 (Lane 2) or anti-c-Myc (Lane 3) were analyzed with Western blotting using anti-V5 and anti-Hh antibodies. These antibodies recognized, respectively, Ptr and Hh contained in the immunocomplex. As expected, the anti c-Myc that was used as the immunoprecipitation control gave no signals.

The response observed in the cell-based reporter gene assays and the observation that Ptr is a transmembrane protein (Pastenes et al., 2008) raise the question of whether Ptr can interact directly with Hh. To test this possibility, we performed an immunoprecipitation assay in which a Ptr-V5 fusion protein was incubated with the conditioned-HhN medium, and the mix was immunoprecipitated with a mouse anti-V5 antibody (for details, see Methods). Co-immunoprecipitated molecules were identified with Western blot analysis using antibodies anti-V5 and anti-Hh. Both Ptr and HhN were identified as forming an immunocomplex, indicating a direct interaction between both proteins (Figure 6). The apparent molecular weights of Ptr and heavy/light chains of anti-V5 were slightly different than predicted (for Ptr-V5, a signal at 120 kDa was predicted, instead of 95 KDa). This biochemical behavior, termed “gel shifting,” might derive from altered binding caused by the detergent employed (Rath et al., 2009; Nybo, 2012). Thus, the data obtained using this method indicated that Ptr was able to bind Hh directly.

### Overexpression of *Ptr* Modifies the Wing A/P Border

**Figure 7:**
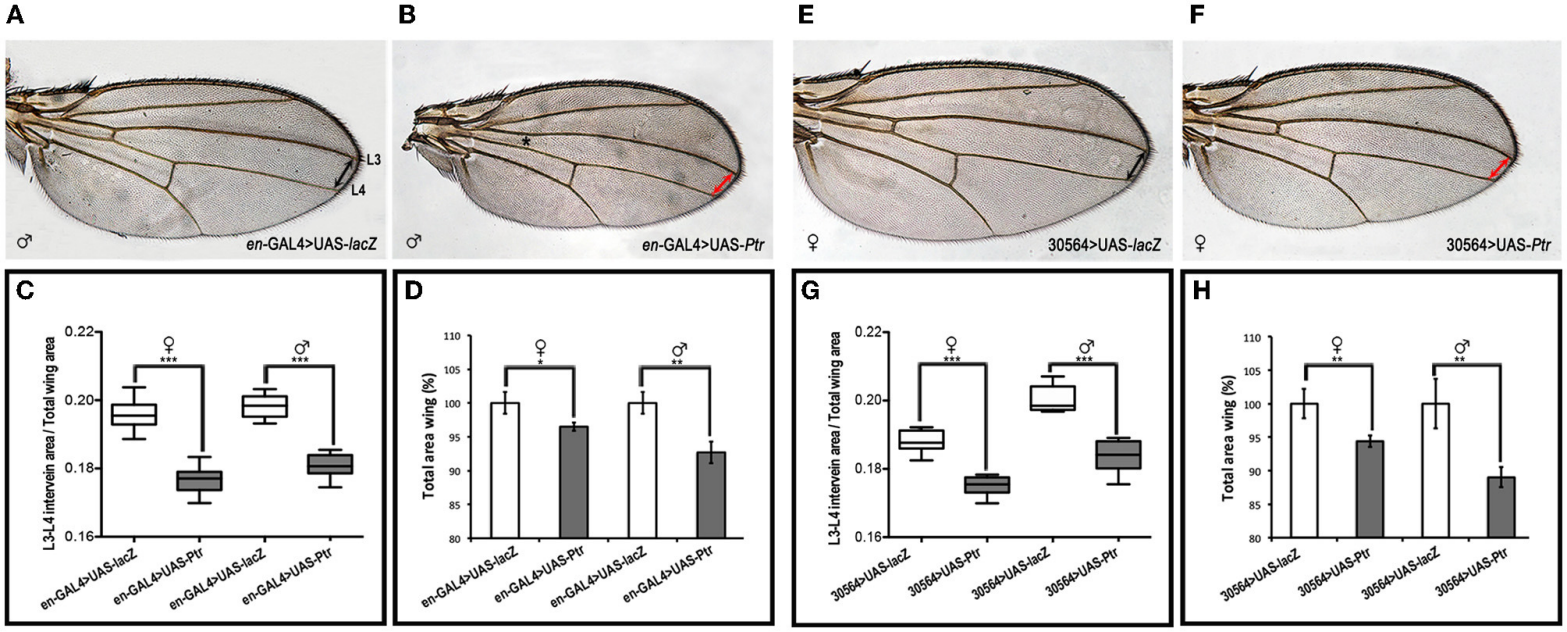
Overexpression of *Ptr* reduces the Hh-signaling-active domain. Wings from male and female adult controls **(A,E)** and from adults expressing UAS-*Ptr* using two different engrailed drivers (*en*-GAL4 and 30564-GAL4, respectively) **(B,F)**. In **(A)**, the longitudinal wing veins 3 and 4 (L3 and L4, respectively) are marked. In **(B)**, the asterisk indicates the location of the missing ACV. In control images, the distance between L3 and L4 is indicated with doubled black arrowheads, whereas, in *Ptr* overexpression wings, this distance is marked with doubled red arrowheads. **(C,G)** The ratio of the L3-L4 intervein area related to the total wing area of each genotype. **(D,H)** Quantification of the whole adult wing area expressed as a percentage of control wings. In all cases, control wings were obtained from *en*-GAL4>UAS-*lacZ* and 30564-GAL4>UAS-*lacZ* crosses (white boxes or columns), while the overexpression wings were obtained from *en*-GAL4>UAS-*Ptr* and 30564-GAL4>UAS-*Ptr* crosses (gray boxes or columns). In each cross, wings from female and male animals were analyzed independently. Analysis was performed using the unpaired two-tailed Student's *t*-test, *p < 0.05, **p < 0.01 and ***p < 0.001.

To further evaluate the participation of Ptr in the Hh pathway, we overexpressed *Ptr* at the wing imaginal disc using a transgenic line that expressed *Ptr*-mCherry under the control of a UAS-activating sequence (Supplementary Figure 3). We used the *en*-GAL4 driver to express *Ptr* not only in the posterior compartment of the imaginal disc but also in a thin strip of cells anterior to the A/P border where *en* and *ptc* coexist (the En/Ptc domain). The signaling occurring in this strip of cells is important for the intervein L3-L4 area and anterior cross vein (ACV) formation (Layalle et al., 2011). As seen in Figure 7, we found that *Ptr* overexpression determined that adult wings have a decreased area between veins 3 and 4 related to the overall wing size. This phenotype was gender independent and reminiscent of that observed when a reduction of Hh activity occurs, since the area between veins L3 and 4 is directly under the control of Hh (Mullor et al., 1997; Strigini and Cohen, 1997; Crozatier et al., 2004). We also noted that the overall size of the wings was reduced, while their general morphology was preserved, indicating that increased Ptr function leads to growth inhibition. In relation to the ACV formation, it has been observed that ≅19% of wings overexpressing Ptr do not present this vein. Similar phenotypes at the A/P border have been previously observed following *ptc* overexpression (Johnson et al., 1995; Martín et al., 2001).

## Discussion

*Ptr* has been identified as one of 118 genes that are differentially expressed in gastrulation (Zúñiga et al., 2009). It is classified as a neuroectodermic gene (Furlong et al., 2001) that encodes an uncharacterized transmembrane protein with a predicted topology closely related to Ptc (Pastenes et al., 2008), the canonical Hh receptor. In this work, using *Ptr* null mutants and the UAS/GAL4 system to direct the expression of *Ptr* dsRNA specifically to neurons, we demonstrated that Ptr is necessary for the proper NS development.

The use of PNA lectin to label primary axonal tracts or pan-neuronal markers, such as anti-elav antibody, indicates that *Ptr* absence or silencing triggers alterations in the NS general morphology. Alterations include distortions in the normal regularity of the spaces between the commissures (anterior and posterior) and the longitudinal tracts, where the axons of the longitudinal tract cross the midline. The occurrence of this kind of malformation has been previously described for *ptc* null mutants (Patel et al., 1989; Merianda et al., 2005), as well as in *hh* overexpression (Bossing and Brand, 2006; Ricolo et al., 2015).

At the antero-posterior level of VNC alterations, *Ptr* null mutants also showed changes in the number and distribution of PNS neurons and axons. These alterations resembled those observed with 22C10 immunostaining of *ptc* mutant embryos, which exhibited similar phenotypes that included the loss of neurons and defects in the organization and pathfinding (Prokopenko et al., 2000). Although experiments are still needed to establish whether peripheral alterations are a consequence of the loss of neurons at the VNC level, it is clear that Ptr is an essential protein for the proper organization of the developing NS.

Ptr is relevant to neuronal function in other species. For instance, *Ptr-18* (one of the 24 *ptr* genes found in *C. elegans*) is essential for establishing the capacity of neural progenitor cells to maintain quiescence in response to nutritional stresses and provides unique insights into the Ptr role in promoting the clearance of extracellular Hh-related protein by targeting it to lysosomal degradation (Chiyoda et al., 2021). Interestingly, PTR-18 is structurally similar to human PTCHD1 (Chiyoda et al., 2021), which has been proposed to cause common neurodevelopmental disorders (Noor et al., 2010). Similarly, in *C. elegans*, PTR-6 participates in the formation of the glial channel that surrounds the receptive endings of the sensory neurons and likely regulates vesicular transport (Perens and Shaham, 2005; Oikonomou et al., 2011; Wallace et al., 2016; Wang et al., 2017).

Results from this work showed that *Ptr* null mutants and knockdown embryos exhibited reduced numbers and altered distribution of Repo-positive glia around the axonal disarray previously mentioned. Although some authors described a reduction in the number of glial cells in *ptc* mutant embryos (Merianda et al., 2005), they dismissed the involvement of glial cells in axonal guidance because the mutant for the *gene glial cell missing* (or *gcm*) employed by the authors does not exhibit a *ptc*-like axon guidance phenotype (Vincent et al., 1996; Takizawa and Hotta, 2001; Merianda et al., 2005). Nevertheless, it will be interesting to investigate whether *Ptr* dsRNA targeting to glial cells causes effects similar to those observed in the null mutant and knockdown embryos, because some aspects of the glial migration are regulated by the same ligand/receptor system that controls the axonal guidance across the CNS midline (Kinrade et al., 2001).

*Ptr* null and knockdown NS alterations also strongly resemble the alterations described in embryos overexpressing *hh* (Bossing and Brand, 2006; Ricolo et al., 2015). In accordance with the proposed function of Ptr in NS development, it has been shown that Hh is involved in several processes of cell migration and guidance (including midline axonal guidance and glial cell migration) by acting as a chemoattractant (direct or indirect) or by regulating cell physiology using both canonical and non-canonical mechanisms (Pielage et al., 2004; Araújo, 2015).

The structural similarities between Ptr and Ptc, together with the association of Ptr with embryo membranes and the resemblance to NS morphological alteration observed in *ptc* null mutants, raised the possibility that Ptr could be involved in the Hh signaling pathway. Interestingly, some reports also associated the *C. elegans* PTR-6 protein with members of the *hh-related* gen family (Aspöck et al., 1999; Oikonomou et al., 2011; Singhal and Shaham, 2017; Wang et al., 2017). Using a cell-based reporter gene assays, we demonstrated that Ptr displays a response characteristic of a negative regulatory component of the pathway, since the RNAi of *Ptr* increased the reporter activity, whereas *Ptr* overexpression suppressed Hh-induced pathway activation. Applying the same experimental approach, we also showed that increased reporter activity produced by *Ptr* dsRNA was enhanced by *ptc* dsRNA, suggesting that, in this cellular system, both receptors can mediate Hh effects. The possibility that cells express receptors with different affinities has been previously suggested by experiments in which a *ptc* allele with low affinity for Hh (Ptc^Con^) was co-expressed with wild-type Ptc in wing imaginal disks (Mullor and Guerrero, 2000). Results indicated that Ptc^Con^ and wild-type Ptc compete for Hh, so a cell containing both receptors could interpret different Hh levels. The difference between affinities implies different states of the transcription factor Cubitus interruptus and subsequent activation of different groups of genes (Mullor and Guerrero, 2000). In our case, the binding of Hh by Ptr could regulate its availability to Ptc, and, as a result, a cell that expresses both Ptr and Ptc could translate the gradient of Hh signaling into a different transcriptional readout when compared to a cell predominantly expressing Ptc. Thus, the possibility of expressing receptors with different or similar affinities for a ligand could enrich the signaling modulation (Yarden and Sliwkowski, 2001; Shibuya and Claesson-Welsh, 2006; Mac Gabhann and Popel, 2008).

In this sense, the results of our luciferase reporter assays suggested that normal levels of Ptr expressed by cl-8 cells are a limiting factor in the response initiated by Hh binding. These results were also consistent with the possibility that Ptr competes with Ptc, limiting Hh signaling. This option is in line with the recent report by Chiyoda et al. (2021) in *C. elegans* where the function of PTR-18 could be linked to the removal of Hh-related extracellular proteins *via* endocytosis-mediated degradation. The same authors speculated that, in *Drosophila*, Ptr might mediate the Ptc-independent Hh internalization. Given that the intracellular Ptr C-terminal contains the same highly conserved motif that is part of the Ptc SSD domain required for Smo translocation to the cell surface (Strutt et al., 2001) and for Ptc endocytosis (Hicke and Dunn, 2003), it would be interesting to know whether Ptr can mediate Hh internalization by itself. Alternatively, Ptr could sequester Hh to deliver it to the vesicular pools of Ptc, similar to the action of Megalin reported in vertebrates (McCarthy et al., 2002). This not only controls the Hh gradient but also regulates the potential of complexing vesicular Ptc with Smo.

In addition to the core components of the Hh signaling pathway in *Drosophil*a, several cell surface proteins have been implicated in modulating the responses to Hh (Beachy et al., 2010), and the existence of an alternative molecule mediating the Hh signaling has been proposed. Although confirmatory experiments are needed, the present results suggested that Ptr could act upstream to Smo and iHog, in spite of the existing literature reporting iHog acting upstream of Ptc (McLellan et al., 2006; Yao et al., 2006; Camp et al., 2014). Therefore, a future challenge will be to investigate the functional interactions between these proteins. Ptr could affect the intracellular trafficking of Smo, given the alternative functions as membrane transporters proposed for proteins structurally related to Ptr (Tseng et al., 1999), or Ptr might function through a non-canonical pathway as had previously reported for Ptc (Brennan et al., 2012; Araújo, 2015; Ricolo et al., 2015).

On the other hand, the binding of Hh to Ptr might require the presence of Brother of ihog (Boi), which is essential for pathway activation but not for Hh reception and sequestration (Camp et al., 2014). It also may need Dally-like (Dlp), a glypican-type heparin sulfate proteoglycan that enhances the stability of Hh and promotes its internalization with Ptc (Yan et al., 2010). Since previous studies have demonstrated that Dlp is specifically required in the cell-based assays and in embryos (Desbordes and Sanson, 2003; Lum et al., 2003; Han et al., 2004), our cell transfection protocols were performed using an expression vector for Dlp (see Methods). Thus, it is possible to speculate that, in the cell assay model we employed, Dlp could facilitate the Ptr-Hh interaction. In spite of this possibility, immunoprecipitation assays between a lysate of cl-8 cells overexpressing Ptr and the concentrated S2-HhN-conditioned medium showed a direct interaction between Ptr and Hh. This result was obtained in conditions that facilitated their interaction, which does not rule out the existence of other molecules that collaborate with Ptr-Hh interaction, mostly when the receptor or the ligand is less available, as occurs *in vivo*.

*In vivo* experiments with *Ptr* overexpression during wing formation confirmed the involvement of Ptr in Hh signaling. Indeed, our data showed that *Ptr* overexpression in the Ptc/En domain of the wing imaginal disks decreased the L3–L4 intervein area, as well as the total wing area and caused the AVC loss in ≅19% of the wings analyzed. All these features have been previously reported for *ptc* overexpression using the same types of drivers (Johnson et al., 1995; McCarthy et al., 2002). Thus, *Ptr* overexpression mimics *ptc* overexpression in the wing imaginal disc and corroborates the participation of Ptr as a negative regulator in the Hh pathway, which includes a possible role in sequestering Hh.

To summarize, our present results showed for the first time that the transmembrane protein Ptr is necessary for the proper NS development and suggested its functional relationship with the Hh pathway. Further *in vivo* studies are needed to explore the role of Ptr in promoting axonal guidance and glial migration, as well as to characterize its direct or indirect interaction with Hh.

## Data Availability Statement

The data will be available upon reasonable request.

## Author Contributions

CB and VC conceived the work. CB, VC, and SO-B wrote the manuscript. CB, SN, and AR performed the fly crosses, assembled the embryo collection, and performed all the procedures related to embryo immunofluorescences. AR and SN determined the hatched rate, obtained, imagined, measured, and analyzed wing-related data. CB and SO-B conducted the confocal microscopy and performed the statistical analysis. CB performed all the procedures related to the cl-8 luciferase reporter, including dsRNA synthesis and cl-8 immunostaining, performed immunoprecipitation and Western blot assays, as well as RNA extraction and qPCR analyses, and generated the article figures. All authors read and approved the final manuscript.

## Funding

This work was supported by the International Brain Organization (IBRO) for its IBRO-LARC PROLAB Program that connected the groups of CB and VC, Comisión Sectorial de Investigación Científica (CSIC I+D 2020 Program, ID 313), and Programa para el Desarrollo de las Ciencias Básicas, MEC-UdelaR (PEDECIBA). SN was supported by CSIC I+D 2020, ID 313.

## Conflict of Interest

The authors declare that the research was conducted in the absence of any commercial or financial relationships that could be construed as a potential conflict of interest.

## Publisher's Note

All claims expressed in this article are solely those of the authors and do not necessarily represent those of their affiliated organizations, or those of the publisher, the editors and the reviewers. Any product that may be evaluated in this article, or claim that may be made by its manufacturer, is not guaranteed or endorsed by the publisher.
