# Supplementary material for: Patched-Related Is Required for Proper Development of Embryonic Drosophila Nervous System
Source: Front Neurosci. 2022 Aug 23;16:920670. doi: 10.3389/fnins.2022.920670 (PMC9446084; doi:10.3389/fnins.2022.920670)
Supplement: Supplementary file 1 [file Data_Sheet_1.docx]

Supplementary Material

**
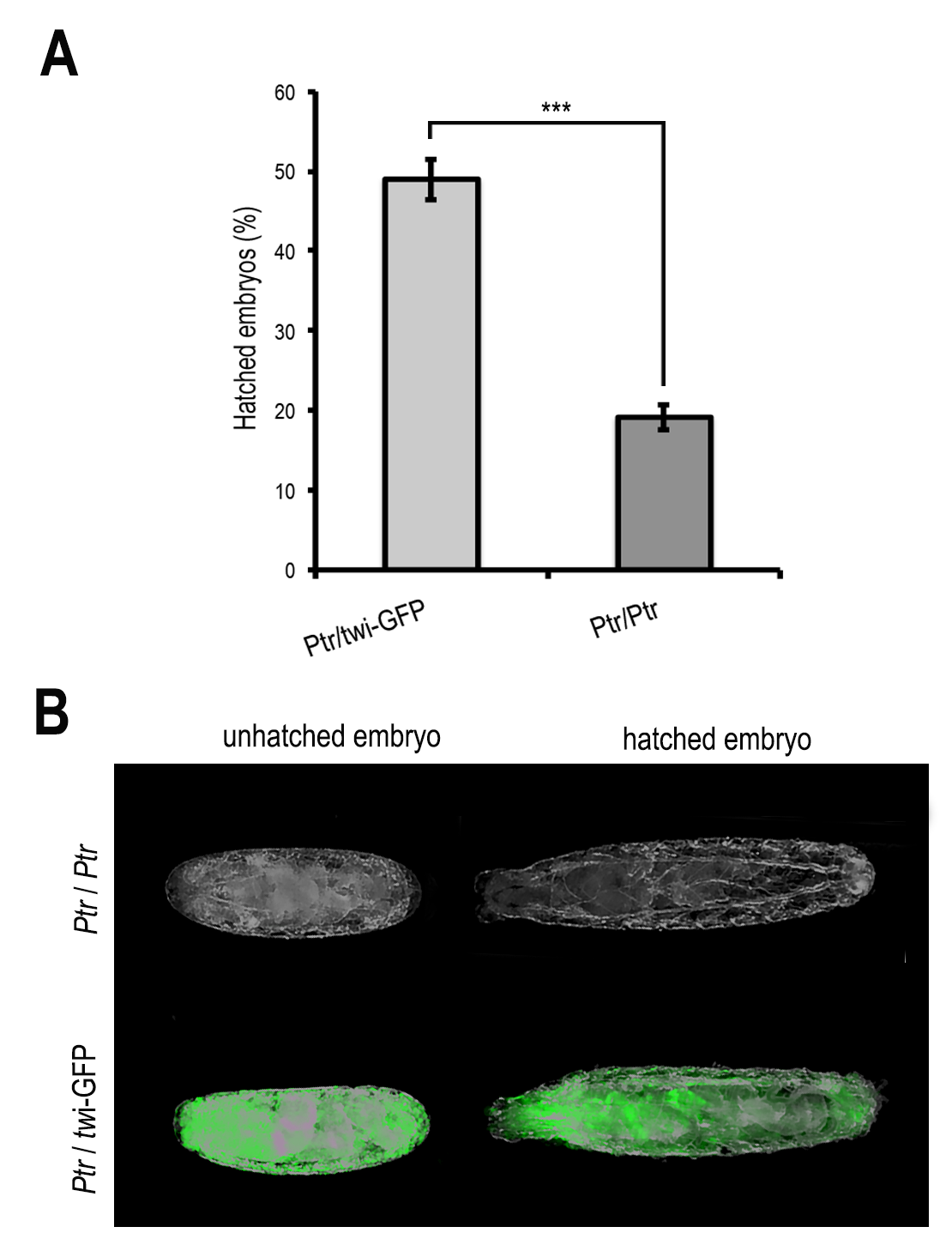
**

**Supplementary Figure 1. Determination of the percentage of unhatched embryos in *Ptr* null mutant line using twist-GFP as balancer. (A)** The percentage of embryos hatching 24 h after egg laying is higher for heterozygous (≈49%) than for homozygous (≈19%) *Ptr* embryos. **(B)** The images show that the fluorescence intensity of *twi*-GFP balancer enables a proper classification of the embryos. Data are presented as mean ± SD (n=3289 embryos were counted during 6 different days). Analysis was performed using unpaired Student *t-*test, ***P<0.001.

**
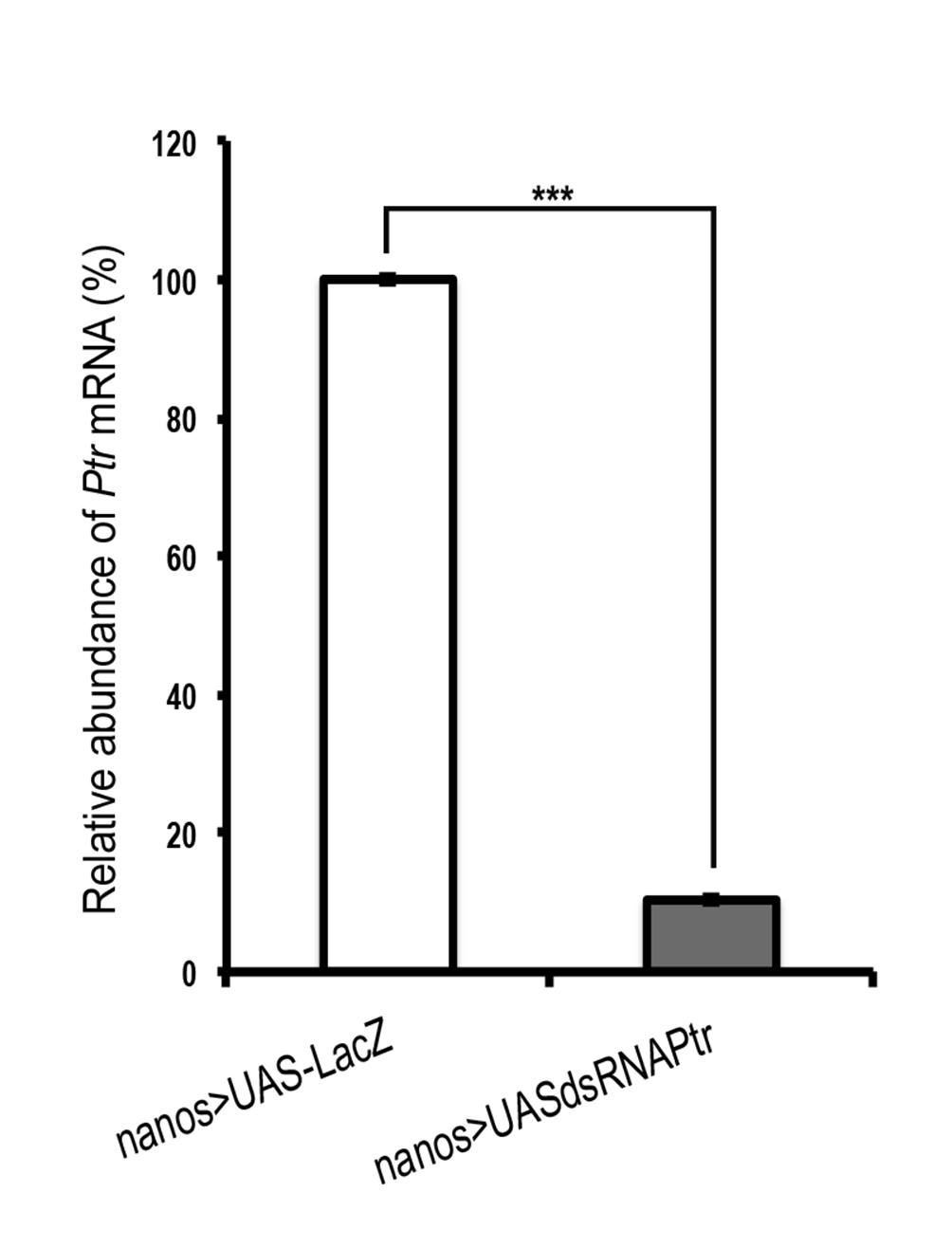
**

**Supplementary Figure 2. Evaluation of UAS-dsRNA Ptr line using nanos-GAL4 driver.** The graphic shows the relative abundance of *Ptr* mRNA in the control and silencing crosses, implying that the construct was effective to down-regulate the *Ptr* gene expression. Data are presented as mean ± SD (n=3 per group). Analysis was performed using unpaired Student *t-*test, ***P<0.001.


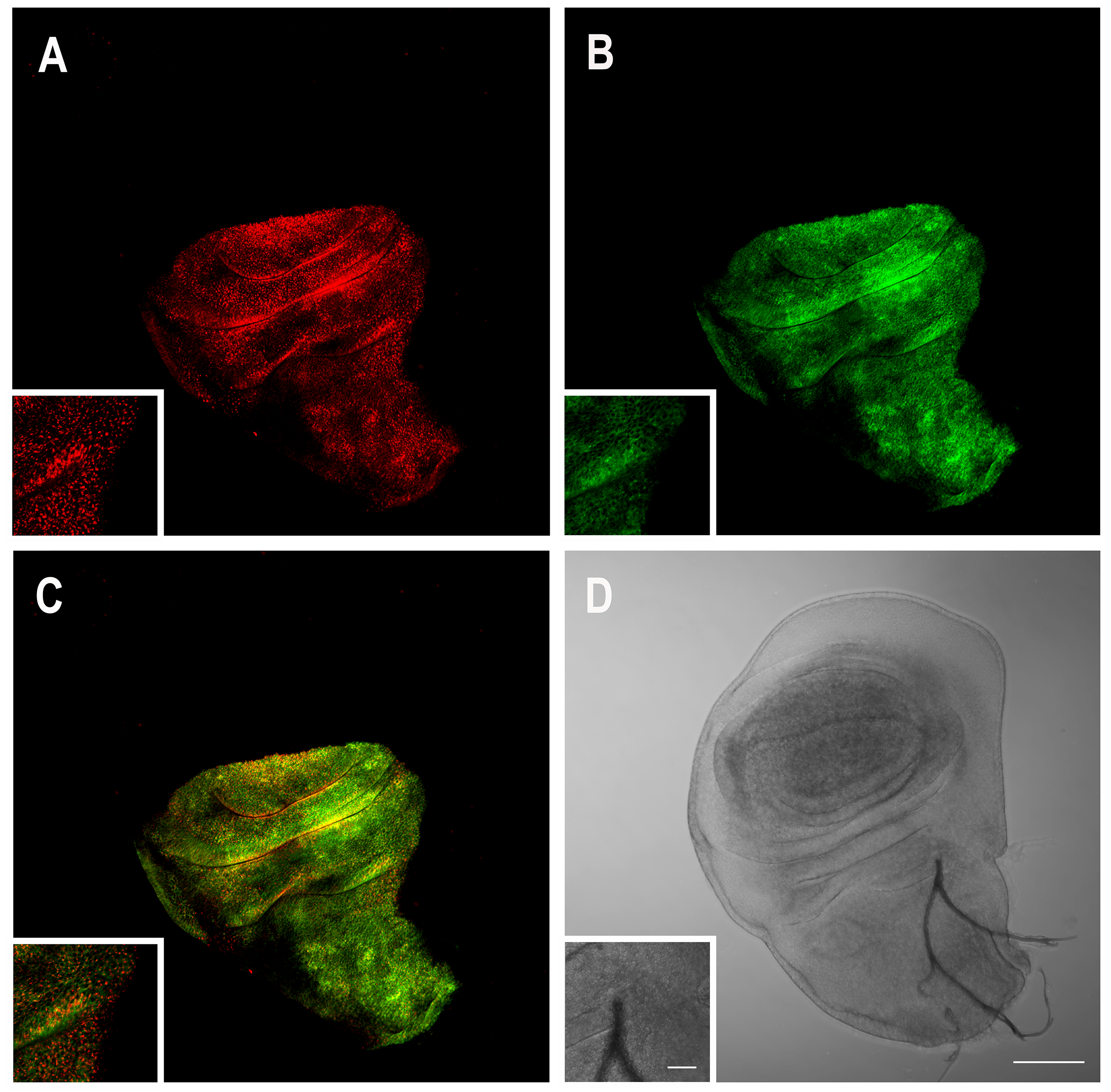


**Supplementary Figure 3.** **Functional evaluation of the *Ptr*-overexpressing line generated in the *Drosophila* imaginal disc**. Ptr-mCherry (red, **A**) is expressed in the dorsal compartment and is monitored by UAS-GFP expression driven by *apterous*-Gal4 (green, **B**). **(C)** Merged images of red and green signals **(D)** Bright field image of an imaginal disc. Insets show higher magnifications to evidence the subcellular distribution of the expressed proteins. Bars: 20 μm (inset) and 100 μm, respectively.
