# Supplementary material for: Patched-Related Is Required for Proper Development of Embryonic Drosophila Nervous System
Source: Front Neurosci. 2022 Aug 23;16:920670. doi: 10.3389/fnins.2022.920670 (PMC9446084; doi:10.3389/fnins.2022.920670)
Supplement: Supplementary file 2 [file Table_1.doc]

**Table of primers**

**Primers used in qPCR and RT-PCR analysis**

| Name | Length (bp) | Tm (°C) | Sequence (5' 3') |
| --- | --- | --- | --- |
| RACE 11212-s1 | 293/233 | 72 | TAAATCGTTTTACCATCTGGGAATATG |
| RACE 11212-a1 |  | 72 | TCGTTATGCGTCCGACGTTAAAGC |
| ptc ex2 ex3 s | 573/236 | 60 | GGACTGTTTCTGGGAGGGA |
| ptc ex2 ex3 a |  | 62 | ATTCAGTGGGTTCAGGCAGG |
| Actin-s | 280 | 62 | CACCGGTATCGTTCTGGACT |
| Actin-a |  | 62 | CTCGTAGGACTTCTCCAACG |
| **Primers used to synthesize dsRNA** | | | |
| Name | Length (bp) | Tm (°C) | Sequence (5' 3') |
| T7-Ex7 Ptr a | 306 | 60 | TAATACGACTCACTATAGGGAGAAACAGACATTGGCTGGACAC |
| T7-Ex7 Ptr s |  | 60 | TAATACGACTCACTATAGGGAGAGTAATGCTGGCAGGTTGG |
| T7-Ex8 Ptr a | 234 | 56 | TAATACGACTCACTATAGGGAGAATTCTGTATGGGAACTCGC |
| T7-Ex8 Ptr s |  | 54 | TAATACGACTCACTATAGGGAGACAAGGACCACATGCTGAT |
| plys-trans-as |  |  | TAATACGACTCACTATAGGGAGACGTAACGGGAAGCATTT |
| plys-trans-s |  |  | TAATACGACTCACTATAGGGAGACGCTGAGGAAGAGGGAC |
| ihog RNAi- s | 637 | 56 | TAATACGACTCACTATAGGGAGACCAAAACCAGCACCACAG |
| ihog RNAi-a |  | 56 | TAATACGACTCACTATAGGGAGAGATTACACGCCAACGCTG |
| ptc RNAi- s | 728 | 58 | TAATACGACTCACTATAGGGAGATGAGCATGCAGATGTCCCT |
| ptc RNAi-a |  | 56 | TAATACGACTCACTATAGGGAGACTAACTCGTAAAGTTATAGCT |
| smo RNAi-s | 709 | 60 | TAATACGACTCACTATAGGGAGAGAATTCCTGCAGAAAAATGGC |
| smo RNAi-a |  | 58 | TAATACGACTCACTATAGGGAGAGCAATAACATTTTGAGTTTGTC |
| ptc exon 5-s | 232 | 60 | TAATACGACTCACTATAGGGAGACCTTCATCTTCTGGGAGCAG |
| ptc exon 5-a |  | 60 | TAATACGACTCACTATAGGGAGACCCACGCTGAGGATGAGTAT |
| ptc exon 6-s | 206 | 60 | TAATACGACTCACTATAGGGA GCCCTTTGAGTTTGTGATCC |
| ptc exon 6-a |  | 60 | TAATACGACTCACTATAGGGACATAGGATTTGCCCGATCTC |
| PEP CG11212-s | 829 | 66 | TAATACGACTCACTATAGGGAGACTCTTCGGACCCGGATCTTGC |
| PEP CG11212-a |  | 66 | TAATACGACTCACTATAGGGAGAGTTGTGGGCAGGCGAGTAAGC |
